# Estimators Used in Multisite Healthcare Costing Studies in Low- and Middle-Income Countries: A Systematic Review and Simulation Study

**DOI:** 10.1016/j.jval.2019.05.007

**Published:** 2019-10

**Authors:** Emma Clarke-Deelder, Anna Vassall, Nicolas A. Menzies

**Affiliations:** 1 Department of Global Health and Population, Harvard T. H. Chan School of Public Health, Boston, MA, USA; 2 Department of Global Health and Development, London School of Hygiene and Tropical Medicine, London, England, UK

**Keywords:** costing, economic evaluation, systematic review, vaccines

## Abstract

**Background:**

In low- and middle-income countries, multisite costing studies are increasingly used to estimate healthcare program costs. These studies have employed a variety of estimators to summarize sample data and make inferences about overall program costs.

**Objective:**

We conducted a systematic review and simulation study to describe these estimation methods and quantify their performance in terms of expected bias and variance.

**Methods:**

We reviewed the published literature through January 2017 to identify multisite costing studies conducted in low- and middle-income countries and extracted data on analytic approaches. To assess estimator performance under realistic conditions, we conducted a simulation study based on 20 empirical cost data sets.

**Results:**

The most commonly used estimators were the volume-weighted mean and the simple mean, despite theoretical reasons to expect bias in the simple mean. When we tested various estimators in realistic study scenarios, the simple mean exhibited an upward bias ranging from 12% to 113% of the true cost across a range of study sample sizes and data sets. The volume-weighted mean exhibited minimal bias and substantially lower root mean squared error. Further gains were possible using estimators that incorporated auxiliary information on delivery volumes.

**Conclusions:**

The choice of summary estimator in multisite costing studies can significantly influence study findings and, therefore, the economic analyses they inform. Use of the simple mean to summarize the results of multisite costing studies should be considered inappropriate. Our study demonstrates that several alternative better-performing methods are available.

## Highlights

• Multisite costing studies are increasingly used to estimate intervention costs in low- and middle-income countries to inform priority setting and economic evaluations. We conducted a systematic review to describe the methods used to produce cost estimates from study data. We then compared the statistical properties of these estimators based on their performance in a simulation study using 20 empirical cost data sets.
• We found substantial variation in the estimation methods used in these costing studies, including the volume-weighted mean (52%), simple mean (34%), and median (21%), and variable reporting of uncertainty estimates. The simulation study showed major differences in the bias and precision of these estimators.
• A substantial fraction of published costing studies reported results based on biased estimators. For those studies reporting the simple mean, cost estimates may be biased upward by 12% to 113%, based on the results of our simulations study. Alternative methods such as the volume-weighted mean will provide unbiased results and should be preferred for use in these studies. More sophisticated methods are available that can produce more precise estimates by leveraging auxiliary information.

## Introduction

In low- and middle-income countries (LMICs), there is increasing interest in understanding the costs of healthcare programs, such as those that deliver vaccines or provide human immunodeficiency virus (HIV) treatment. Cost data can be used to prioritize health interventions, inform budgeting and planning for the scale-up of health programs, and identify opportunities for improved efficiency.^1^, ^2^, ^3^, ^4^ Cost data can also inform planning for sustainable health financing as countries grow economically and transition out of support from international donors such as Gavi, the Vaccine Alliance, and the Global Fund.^5^, ^6^ In recent years, several large multisite costing studies have been conducted to improve the availability of cost data for global health programs when routine data are unavailable. These studies are distinguished by their analyses of cost and service volume data collected from a sample of healthcare delivery sites, with the aim of drawing conclusions about the larger program in which the study sites operate. These studies typically have reported summary estimates for the costs per person or for the total costs of the overall program.

Multisite healthcare costing studies have adopted different approaches to estimating program-level costs using data from a sample of sites. For example, a study analyzing data from 161 HIV treatment sites across 5 countries reported both the simple and the volume-weighted mean of facility-level unit costs for each country in the study.^7^ A Centers for Disease Control–supported costing study of HIV treatment in programs supported by the United States President's Emergency Plan for AIDS (PEPFAR) used the median to create country-specific estimates using cost data from 43 sites across 5 countries.^3^ Researchers reporting the results of the EPI costing & financing project, a study of routine infant immunization costs across 319 sites and 6 countries, used a variety of methods to summarize results, including both simple and volume-weighted means.^8^, ^9^, ^10^, ^11^, ^12^, ^13^

Estimation techniques differ in terms of the quality of inference they provide. The statistical characteristics of an estimator can be summarized by its bias (the difference between the expected value of the estimator and the true value being estimated) and efficiency (the variance of the estimator in comparison with other possible estimators). Estimation errors (differences between the estimate and the true quantity) can influence the conclusions drawn from multisite healthcare costing studies and the economic analyses they inform.

Given the variation in estimation methods across multisite healthcare costing studies in LMICs, there is a need to better understand the benefits and drawbacks of different approaches. The theoretical properties of estimators for ratios (such as the costs per person of a healthcare program) and totals (such as the total costs of a healthcare program) have been well studied in the sample statistics literature.^14^, ^15^ Quantitative measures of estimator performance (such as the magnitude of bias) depend on the characteristics of the data being studied. For example, the presence and magnitude of economies of scale in healthcare programs in LMICs^16^, ^17^ are likely to influence the performance of different summary estimators. In this article, we describe current estimation practices in the multisite healthcare costing literature in LMICs, compare different estimation methods, and make recommendations for calculating and presenting summary statistics in these studies.

## Methods

### Systematic review

We conducted a preregistered systematic review of the summary estimators used in published multisite costing studies from LMICs. We searched Pubmed for studies published through January 1, 2017, using terms related to costs and LMICs, and similarly searched an online costing study repository.^18^ Search strategies are described in the supplementary appendix (see Appendix Table 1 in Supplementary Materials found at https://doi.org/10.1016/j.jval.2019.05.007). We included studies that (1) analyzed cost data from 5 or more sites in an LMIC, as defined by the World Bank in 2016; (2) considered a defined individual health intervention, package of interventions, or care for a defined health condition; (3) reported a summary estimate of central tendency for total or unit costs of the health intervention, and (4) were published in English. We excluded studies based on reported charges such as claims data sets, studies reporting only site-level costs without any summary estimate, and studies of community-level interventions, such as behavior change campaigns. We did not consider composite cost estimates combining data from multiple health system levels because the methodological issues around these estimates were beyond the scope of this study. One author screened titles and abstracts to identify articles for full text review and then reviewed full text articles to determine inclusion and extract relevant data. From each included study we extracted bibliographic information, information on health intervention and setting, and estimation approach. We analyzed the characteristics of the included studies and the proportion of studies that included different summary estimators. We disaggregated findings by publication year and health intervention.

### Evaluation of estimator properties

To evaluate estimator performance, we imputed realistic complete population cost data sets for different health programs using empirical sample data from costing studies in LMICs. The imputed data sets contained cost and service delivery volume information for all healthcare delivery sites in each program (eg, all routine immunization delivery sites in Honduras). From each imputed data set, we drew repeated samples of varying sizes and compared how different estimators performed in estimating the unit costs of delivery in the overall program.

#### Imputation of complete population data sets

Quantitative descriptions of estimator performance require realistic information about the data being studied. To create realistic data sets of full populations of healthcare delivery sites, we imputed data sets of healthcare delivery costs and volumes using information from the 20 empirical data sets listed in Table 1. The original empirical data sets, which were publicly available or were shared by the original study authors, contained data on a sample of service delivery sites and represented 12 countries (Benin, Ethiopia, Ghana, Honduras, India, Kenya, Malawi, Moldova, Rwanda, South Africa, Uganda, Zambia) and 4 types of health interventions (HIV prevention, HIV testing, HIV treatment, and routine immunization).^7^, ^8^, ^9^, ^10^, ^12^, ^13^, ^19^, ^20^, ^21^ They therefore reflected variation across countries and health interventions in the distribution of costs and service delivery volumes. We included only data sets with 30 or more delivery sites because these larger studies provide better evidence for imputing realistic populations of sites (Table 1).

**Table 1:** Empirical data sets used in our simulation study

Original study	Health intervention	Sampling frame available	Country	Number of sites
Avahan^20^	HIV prevention	No	India	129
EPIC^8^, ^9^, ^10^, ^11^, ^12^, ^13^	Routine immunization	Yes	Benin	46
			Ghana	50
			Honduras	71
			Moldova	50
			Uganda	49
			Zambia	51
MATCH^7^	HIV treatment	Yes	Ethiopia	41
			Malawi	30
			Rwanda	30
			Zambia	30
ORPHEA^19^	Prevention of mother-to-child transmission of HIV	No	Kenya	51
			Rwanda	53
			South Africa	42
			Zambia	56
ORPHEA^19^	HIV testing and counseling	No	Kenya	56
			Rwanda	53
			South Africa	42
			Zambia	60
Marseille et al.^21^	HIV treatment	No	Zambia	45

EPIC indicates the multi-country study on the costing and financing of routine immunization and new vaccines; HIV, human immunodeficiency virus; MATCH, Multi-Country Analysis of Treatment Costs for HIV/AIDS; ORPHEA, Optimizing the Response in Prevention: HIV Efficiency in Africa.

When a study's sampling frame was available and had full information on delivery volume in the population, we used this sampling frame to impute cost data for nonsampled sites. When these data were not available, we imputed both volumes and costs for nonsampled sites. We used nonparametric approaches for imputation to most accurately reflect the empirical distributions of costs and volumes in the observed data and to avoid making parametric assumptions that might mirror those used in later analysis. To impute volume data, we sampled from a kernel density fit to the distribution of delivery volumes in the sample.^22^ To impute cost data, we first used a regression spline to estimate the mean relationship between costs and volume in the empirical sample and then included dispersion around this mean relationship by sampling from the distribution of residuals from the fitted regression. This approach is similar to a parametric bootstrap but relaxes the distributional assumptions around the residuals from this approach (eg, allowing for outliers).^23^ We compared the imputed data with the empirical samples to confirm that the imputation approach reproduced the features of the sample. Our imputation methods are described further in the supplementary appendix (See Appendix 1.2 in Supplementary Materials found at https://.doi.org/10.1016/j.jval.2019.05.007).

#### Simulation of repeated multisite cost studies

From each imputed data set, we drew 10,000 simple random samples, each of sizes 5, 10, 20, 40, and 80. We analyzed each sample using 5 estimators of central tendency: the simple mean, the volume-weighted mean, the median, a calibration estimator that reweights the sample to reproduce known features of the population (in this case, total delivery volume), and a regression estimator using a log-log specification. The first 3 estimators were the most commonly used in the reviewed literature. The calibration and regression estimators were not found in the literature but were included to assess whether they might perform better than the commonly used estimators. We did not examine the full universe of estimators, but the 5 that were selected reflect the most commonly used estimators and a subset of the possible alternatives with the potential to outperform the more commonly used approaches. Both the calibration and regression estimators use auxiliary information to improve estimation: the calibration estimator uses the total service delivery volume in the program of interest, and the regression estimator uses the service delivery volume of every site in the program of interest. This information may be available, as it is often used to inform sampling procedures.

The five estimators were implemented as follows, where *n* represents the number of sites in the sample, *C*_*i*_ represents the total service delivery costs at site *i*, and *Q*_*i*_ represents the total service delivery volume at site *i*:

• *Simple mean:* The simple mean was calculated as follows:

$$Mean_{s}=\frac{1}{n}\sum_{i=1}^{n}\frac{C_{i}}{Q_{i}}\;$$

• *Volume-weighted mean:* The volume-weighted mean was calculated as follows:

$$Mean_{vw}=\frac{\sum_{i=1}^{n}C_{i}}{\sum_{i=1}^{n}Q_{i}}$$

• *Median:* The median was calculated as the median of the site-level unit costs in the sample, using the “median” function in R.^24^
• *Calibration estimator:* The calibration estimator reweights sample values such that the total service volume implied by the weighted sample is equal to the total volume in the population.^25^, ^26^ Using the Newton-Raphson algorithm, the weights are adjusted such that the sum of the weighted sample volumes equals true total volume. This estimator was implemented using the *survey* package in R,^27^ as previously described elsewhere.^28^
• *Regression estimator:* A linear regression model of the following form was fit to the sampled data:

$$\log\left(C_{i}\right)=\alpha +\beta \text{log}\left(Q_{i}\right)+\varepsilon_{i}$$

This model was then used to make predictions for the total costs at each site in the overall population. Unit costs were estimated as the sum of the predicted costs divided by the (known) total delivery volume in the population.

We calculated the bias, variance, and root mean squared error (RMSE) of each estimator in each data set for each sample size. We calculated the bias as the difference between the expected value of the estimator (over repeated samples from a particular population) and the true value of the parameter (in that population). RMSE, which is the square root of the average squared deviation of the estimator from the true parameter value, combines information about bias and variance into a single measure (where the squared RMSE is the sum of the variance and the squared bias). We focused on identifying the estimator that minimized RMSE.

Results are reported by intervention area, then pooled across all data sets. In the pooled analysis, we examined (1) the average performance across different imputed data sets, as measured by bias, standard deviation, and RMSE, and (2) the frequency of extreme results, as measured by the proportion of estimates greater than a specified percentage away from the true parameter value. For comparability, bias, standard deviation, and RMSE are reported as a percentage of the true unit cost for each data set.

#### Sensitivity analyses

By using empirical data sets from different health domains in our simulation study, we were able to study estimator performance in a range of data sets with realistic cost-volume relationships. For a sensitivity analysis, we also examined how bias varied when changing the costvolume relationship or the amount of variation in delivery volumes.

We drew simple random samples in our main analysis. For sensitivity analyses, we also compared estimator performance under stratified random sampling and sampling proportional to volume.

The Institutional Review Board of the Harvard T. H. Chan School of Public Health determined that this study was not human-subjects research.

## Results

### Systematic review

We identified 6774 records for initial review. After applying our inclusion criteria, we identified 100 studies for analysis (see Appendix Fig. 1 in Supplementary Materials found at https://doi.org/10.1016/j.jval.2019.05.007). Table 2 summarizes findings from the systematic review. We found that the number of studies meeting inclusion criteria increased significantly in recent years: We identified 61 eligible studies in the 2012 to 2016 period and only 23 in the 2007 to 2011 period. Before 2007, this type of study in LMIC settings was rare.

**Table 2:** Summary of findings from our systematic review∗

	Number of publications (%), by year		
	Before 2007	2007-2011	2012-2016
All	16	23	61
Region (World Health Organization)	Before 2007	2007-11	2012-16
Africa	6 (38)	12 (52)	39 (64)
Americas	3 (19)	5 (22)	8 (13)
Eastern Mediterranean	0	0	3 (5)
Europe	0	0	2 (3)
Southeast Asia	6 (38)	8 (35)	10 (16)
Western Pacific	1 (6)	1 (4)	3 (5)
Health domain	Before 2007	2007-11	2012-16
HIV/AIDS	6 (38)	7 (30)	30 (49)
Vaccination	5 (31)	1 (4)	7 (11)
Reproductive and maternal health	3 (19)	3 (13)	12 (20)
Malaria	0	4 (17)	3 (5)
Other	2 (13)	8 (35)	9 (15)
Number of delivery sites (total, across all countries in study)	Before 2007	2007-2011	2012-2016
5-9	2 (13)	5 (22)	16 (26)
10-14	2 (13)	6 (26)	8 (13)
15-19	7 (44)	6 (26)	6 (10)
20+	5 (31)	6 (26)	30 (49)
Not reported	0	0	1 (2)
Site sampling approach	Before 2007	2007-2011	2012-2016
Simple random sampling	2 (13)	1 (4)	4 (7)
Stratified random sampling	7 (44)	3 (13)	14 (23)
Clustered random sampling	3 (19)	6 (26)	11 (18)
Stratified and clustered random sampling	2 (13)	0	6 (10)
Purposive sampling	6 (38)	15 (65)	34 (56)
Exhaustive sampling	0	3 (13)	12 (20)
Sampling approach not described	0	2 (9)	0
Outcome measured	Before 2007	2007-2011	2012-2016
Total costs (eg, for country or district)	1 (6)	7 (30)	14 (23)
Cost per site	5 (31)	7 (30)	16 (26)
Cost per person, service, or person-time of care	15 (94)	22 (96)	57 (93)
Other	4 (25)	3 (13)	1 (2)
Reported variation in site-level cost estimates	Before 2007	2007-2011	2012-2016
Range	4 (25)	5 (22)	11 (18)
Interquartile range	0	2 (9)	5 (8)
Standard deviation	0	2 (9)	8 (13)
All sampled values reported	4 (25)	2 (9)	7 (11)
Other	1 (6)	1 (4)	0
Not reported	8 (50)	12 (52)	37 (61)
Summary estimator of central tendency	Before 2007	2007-2011	2012-2016
Simple average across sites	5 (31)	10 (43)	19 (31)
Average across sites, weighted by volume	10 (63)	13 (57)	24 (39)
Average across sites, weighted by other characteristics	2 (13)	2 (9)	3 (5)
Average across sites, weighted by volume and other characteristics	0	1 (4)	5 (8)
Median across sites	4 (25)	6 (26)	11 (18)
Simple average across individuals, sampled from multiple sites	0	3 (13)	6 (10)
Other	5 (31)	7 (30)	8 (13)
Not described	0	1 (4)	11 (18)
Estimate of uncertainty in central tendency	Before 2007	2007-2011	2012-2016
Reported	1 (6)	2 (9)	9 (15)
Not reported	15 (94)	21 (91)	52 (85)

∗ Publications may be counted twice if they fit into more than 1 of the categories listed under a given heading. For example, a publication may be counted twice under the “Region” heading if it includes data from an African country and from a Southeast Asian country.

A variety of estimators were used to summarize cost data. Approximately one-third of the studies (37.0%) reported more than 1 summary estimate of costs. The most commonly reported summary estimators were the volume-weighted mean (52.0% of studies) and the simple mean (34.0%). Of the studies analyzed, 13.0% exclusively reported the simple mean and 14.0% reported both the simple mean and the volume-weighted mean.

Most studies reported only summary estimates and did not report the variance or any other characteristics of the distribution of site-level estimates. Only 12% of studies reported confidence intervals or other measures to describe statistical uncertainty in summary estimates (Table 2).

As shown in Table S2 (see Appendix Table 2 in Supplementary Materials found at https://doi.org/10.1016/j.jval.2019.05.007), estimator use varied by health domain. The simple mean was reported in 32.6% of HIV studies, 30.8% of vaccination studies, and 57.1% of malaria studies in the review. The volume-weighted mean (or mean weighted by volume and other characteristics) was reported in 58.1% of HIV studies, 61.5% of vaccination studies, and 42.9% of malaria studies in the review.

### Estimator bias, variance, and RMSE

**Figure 1:**
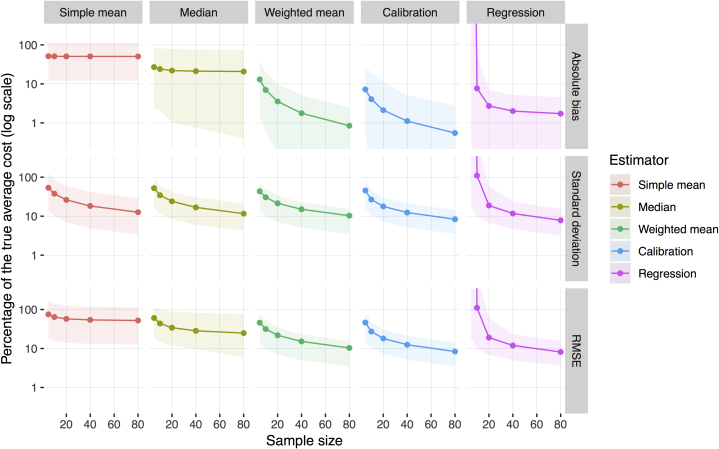
Estimator performance as measured by absolute bias, standard deviation, and root mean squared error (RMSE) for 5 estimators across all included studies (log scale). Each column of panels shows the results for a particular estimator (labeled across the top of the figure). Each row of panels shows results for a given measure of estimator performance. The x-axis is the sample size used in the simulation. The y-axis is the measure of estimator performance (estimated through simulation), presented as a percentage of the true unit cost, on the log scale. The ribbons represent the range of results from conducting simulations in different imputed data sets: The bottom of the ribbon is the best result, the top is the worst result, and the solid line is the mean result across data sets. For example, the upper far left panel can be interpreted as follows: On average across the different data sets in our simulation, the simple mean estimator has an absolute bias of 51% of the true cost in samples of 5 sites (range from 12%-113%). This bias remains roughly constant for increasing sample sizes. In contrast, the upper far right panel shows that the absolute bias in the regression estimator is very large in samples of 5 sites and decreases to 9% on average (range 0.1%-54%) in samples of 10 sites, 3% on average (range 0.0%-7%) in samples of 20 sites, 2% on average (range 0.2%-5%) in samples of 40 sites, and 2% on average (range 0.2%-5%) in samples of 80 sites.

Figure 1 shows the mean performance of each estimator across the 20 data sets in the simulation, bounded by the best and worst performance of that estimator. The top row of panels shows that whereas the bias in the simple mean and median stayed large as the sample size increased, the bias of the other estimators decreased with sample size. In samples of 80 sites, the simple mean exhibited an absolute bias of 50.6% and the median exhibited an absolute bias of 20.8% of the true unit cost on average across data sets. The simple mean had a positive bias in all 20 data sets. The median had a positive bias in 18 of 20 data sets.

As shown in the middle row of panels, the standard deviation of the different estimators varied slightly. In samples of 40 or 80 sites, the calibration and regression estimators generally outperformed other estimators.

The bottom row of panels shows the RMSE of each estimator for different sample sizes. In samples of 5 sites, the weighted mean and calibration estimator had the lowest RMSEs (45.9% and 46.3% of the true parameter value, respectively). In samples of 10 or 20 sites, the calibration estimator had the lowest RMSE (18.1%-27.3%). In samples of 40 or 80 sites, the regression estimator had the lowest RMSE (8.1%-12.0%) (Fig. 1).

**Figure 2:**
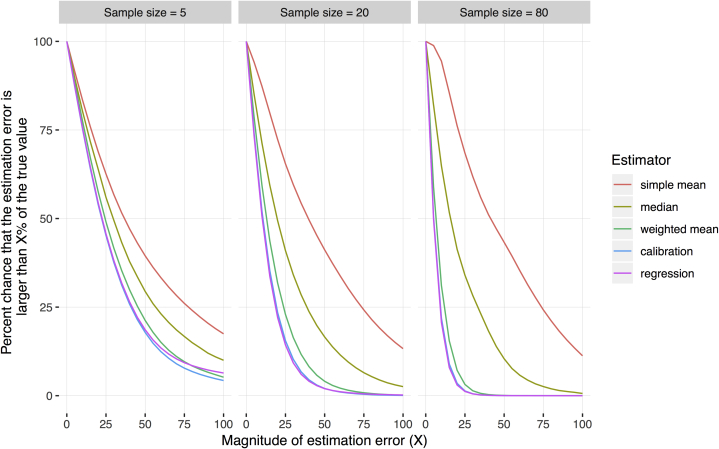
Percent chance of estimation error larger than a specified value. Each panel shows results from simulations of a different sample size (5, 20, and 80 sites). The lines show findings for 5 estimators. The y-axis is the probability that an estimate (generated by a particular estimator with samples of a particular size) will differ from the true population value by more than the percentage (X) along the x-axis. For example, in the middle panel, the place where the simple mean line crosses the 50% line on the x-axis can be interpreted as follows: For sample sizes of 20 sites, the simple mean generates estimates that are more than 50% away from the true value approximately 43% of the time.

Figure 2 shows the fraction of samples in which the estimated value differed from the true value by more than a specified percentage. As the sample size increased, the calibration estimator, weighted mean, and median all performed noticeably better, but the simple mean did not. In our simulation, in samples of 20 sites, there was a 13.3% chance that the simple mean would generate estimates that were more than 100% away from the true unit cost. There was a 41.2% chance that the simple mean would generate estimates that were more than 50% away from the true unit cost. Even in samples of 80 sites, there was an approximately 11.3% chance that the simple mean would generate estimates that were more than 100% away from the true value. In contrast, in samples of 20 sites, there was an only 2.0% chance that the calibration or regression estimators would generate estimates more than 50% away from the true unit cost, and there was only a 4.1% chance that the weighted mean would generate estimates more than 50% away from the true unit cost (Fig. 2).

We expect the performance of the simple mean to be influenced by the strength of the relationship between costs and volume and by the variance in delivery volumes. In a sensitivity analysis, we examined the empirical relationship between these characteristics and the bias estimates from our main analysis. The results in Figure S4 (see Appendix Fig. 4 in Supplementary Materials found at https://doi.org/10.1016/j.jval.2019.05.007) confirm that bias in the simple mean increases with the strength of the cost-volume relationship and with the variation in delivery volumes in the population. A regression of estimated bias on coefficient of variation and elasticity estimates for each study sample showed that a single standard deviation increase in the coefficient of variation in delivery volumes was associated with a 24.5% (95% confidence interval [CI] 14.3-34.9) increase in the bias. A single standard deviation increase in the magnitude of the elasticity with respect to delivery volumes (ie, an increasingly negative elasticity) was associated with a 62.0% (95% CI 50.4-74.7) increase in the bias in the simple mean.

We also examined how estimator performance changed with different sampling approaches. Under stratified sampling, our main conclusions do not change. Under sampling proportional to delivery volume, the bias in the simple mean is resolved. With this approach, weighting is done in the sampling stage rather than in the analysis stage. Results from sensitivity analyses are reported in the supplementary appendix (See Appendix 2.2.2 in Supplementary Materials found at https://doi.org/10.1016/j.jval.2019.05.007).

## Discussion

There are many potential sources of variation in healthcare cost estimates, including the way researchers conceptualize the appropriate cost data to collect and the data collection methods they use (eg, retrospective or prospective, top-down or bottom-up approaches). Another important source of variation in multisite cost studies results from the techniques used to analyze data. The question of how to select an appropriate sample has been explored extensively elsewhere^14^, ^15^; this study focused on comparing methods for estimating central tendency after a sample has been collected. We reviewed the literature of multisite healthcare costing studies in LMICs to understand the estimators used to generate summary estimates. We then conducted computer experiments to assess the performance of 5 summary estimators in realistic scenarios created from a large number of empirical healthcare costing data sets. We found substantial variation in the methods used to summarize data collected from a sample of delivery sites and found large and avoidable deficiencies with some of these methods.

Our systematic review identified 100 studies published through the end of 2016, the majority of which were published from 2012 to 2016. Across these studies, the most commonly used summary estimators were the volume-weighted mean, the simple mean, and the median. The simple mean, which was shown in our simulations to overestimate unit costs by 50.6% on average, was reported in 34.0% of the studies included in the review.

Evaluations of estimator performance are often particularly concerned with unbiasedness and asymptotic properties. However, our systematic review highlighted that multisite costing studies tend to have small samples. In this setting, variance is an important contributor to estimation error. Because the main goal in selecting an estimator is to reduce overall error, we used RMSE to compare the estimators in our study.

In simulations, the calibration estimator, regression estimator, and volume-weighted mean all performed significantly better than the simple mean or median in terms of RMSE. Each of these 3 estimators has advantages. The volume-weighted mean had the largest RMSE of the 3 estimators, but it requires the least data, is the least computationally complex, and may be the simplest method to describe to stakeholders. For these reasons, in many studies it may be preferred over the calibration or regression estimator. Both the calibration estimator and the regression estimator improve on the performance of the simple mean by leveraging auxiliary information. This reduces variance relative to the simple mean, but it also requires additional data: The regression estimator requires data on site-level volumes for all sites, and the calibration estimator requires data on the total number of sites and total service volume. Therefore, when the 3 best-performing estimators are compared, the volume-weighted mean requires the least auxiliary information, followed by the calibration estimator and then the regression estimator. In situations where it is not feasible to collect data from a large number of sites, it may be advantageous to collect auxiliary information on volumes that can allow the use of a regression or calibration estimator to reduce variance. Although we did not examine this in our analysis, the calibration and regression estimators could both incorporate additional information beyond delivery volume (eg, facility type, case mix, or quality). The regression estimator could also be used with alternative specifications. Complex functional forms or additional predictors could, however, lead to overfitting, resulting in worse predictive performance.

Our study has several limitations. Our simulations did not include all possible methods but, rather, focused on the main methods used in the literature and several viable alternatives. We simulated simple random sampling, but a large proportion of the studies included in our systematic review used purposive sampling to select health facilities. Although we cannot know how this influences results, if purposive selection is informed by relevant characteristics such as facility size (ie, if it functions as a crude form of stratified sampling), then the performance of all of the included estimators could improve relative to simple random sampling.

Although our main findings are consistent across data sets from different health domains, estimator performance will vary depending on the characteristics of the health program being studied. For example, the bias in the simple mean will tend to be lower if large portions of the cost of a healthcare program exhibit no economies of scale (eg, medications) and higher if large portions of the cost exhibit significant economies of scale (eg, human resource costs).

## Conclusion

The choice of summary estimator in multisite costing studies can significantly influence study findings and, therefore, the economic analyses they inform. For researchers who use the results from costing studies in economic models, our findings highlight the importance of considering potential bias in cost estimates. Use of the simple mean to summarize the results of multisite costing studies should be considered inappropriate. Our study demonstrates that several alternative better-performing methods are available.

## Acknowledgments

We thank Sergio Bautista-Arredondo, Jim Kahn, Elliot Marseille, and Maaya Sundaram for sharing data for use in the simulation study and Rifat Atun, Logan Brenzel, Sebastian Haneuse, Stephen Resch, Stephane Verguet, and Alan Zaslavsky for providing comments on earlier versions of this article.

This study was funded by the Bill & Melinda Gates Foundation (Grant # OPP1158709).
