# Supplementary material for: Estimators Used in Multisite Healthcare Costing Studies in Low- and Middle-Income Countries: A Systematic Review and Simulation Study
Source: Value Health. 2019 Oct;22(10):1146–53. doi: 10.1016/j.jval.2019.05.007 (PMC6859917; doi:10.1016/j.jval.2019.05.007)
Supplement: Supplementary Materials [file mmc1.docx]

**Systematic review and simulation study of estimators used in multi-site healthcare costing studies in low- and middle-income countries: Supplementary appendix**

[1 Supplementary methods 1](#_Toc511228526)

[1.1 Systematic review 1](#_Toc511228527)

[1.2 Simulation model 2](#_Toc511228528)

[1.2.1 Imputation of delivery volume 2](#_Toc511228529)

[1.2.2 Imputation of costs 3](#_Toc511228530)

[1.2.3 Comparison of imputed populations with sample data 3](#_Toc511228531)

[2 Supplementary results 5](#_Toc511228532)

[2.1 Systematic review 5](#_Toc511228533)

[2.1.1 Studies included 5](#_Toc511228534)

[2.2 Simulation model 14](#_Toc511228535)

[2.2.1 Estimator performance 14](#_Toc511228536)

#

# **Supplementary methods**

## **Systematic review**

Table S1: Electronic search strategies used

| **Database** | **Search strategy** |
| --- | --- |
| Pubmed | Search terms for PubMed included: ("cost"[ti] OR "costs"[ti] OR "costing"[ti]) AND ("Afghanistan"[tiab] OR "Albania"[tiab] OR "Algeria"[tiab] OR "Samoa"[tiab] OR "Angola"[tiab] OR "Argentina"[tiab] OR "Armenia"[tiab] OR "Azerbaijan"[tiab] OR "Bangladesh"[tiab] OR "Belarus"[tiab] OR "Belize"[tiab] OR "Benin"[tiab] OR "Bhutan"[tiab] OR "Bolivia"[tiab] OR "Bosnia"[tiab] OR "Botswana"[tiab] OR "Brazil"[tiab] OR "Bulgaria"[tiab] OR "Burkina Faso"[tiab] OR "Burundi"[tiab] OR "Cabo Verde"[tiab] OR "Cape Verde"[tiab] OR "Cambodia"[tiab] OR "Cameroon"[tiab] OR "Central African Republic"[tiab] OR "CAR"[tiab] OR "Chad"[tiab] OR "China"[tiab] OR "Colombia"[tiab] OR "Comoros"[tiab] OR "DRC"[tiab] OR "Congo"[tiab] OR "Zaire"[tiab] OR "Costa Rica"[tiab] OR "Cote d'Ivoire"[tiab] OR "Ivory Coast"[tiab] OR "Cuba"[tiab] OR "Djibouti"[tiab] OR "Dominica"[tiab] OR "Dominican Republic"[tiab] OR "Ecuador"[tiab] OR "Egypt"[tiab] OR "El Salvador"[tiab] OR "Equatorial Guinea"[tiab] OR "Eritrea"[tiab] OR "Ethiopia"[tiab] OR "Fiji"[tiab] OR "Gabon"[tiab] OR "Gambia"[tiab] OR "Georgia"[tiab] OR "Ghana"[tiab] OR "Grenada"[tiab] OR "Guatemala"[tiab] OR "Guinea"[tiab] OR "Guinea-Bissau"[tiab] OR "Guyana"[tiab] OR "Haiti"[tiab] OR "Honduras"[tiab] OR "India"[tiab] OR "Indonesia"[tiab] OR "Iran"[tiab] OR "Iraq"[tiab] OR "Jamaica"[tiab] OR "Jordan"[tiab] OR "Kazakhstan"[tiab] OR "Kenya"[tiab] OR "Kiribati"[tiab] OR "Korea"[tiab] OR "Kosovo"[tiab] OR "Kyrgyz"[tiab] OR "Kyrgyzstan"[tiab] OR "Lao"[tiab] OR "Laos"[tiab] OR "Lebanon"[tiab] OR "Lesotho"[tiab] OR "Liberia"[tiab] OR "Libya"[tiab] OR "Macedonia"[tiab] OR "Madagascar"[tiab] OR "Malawi"[tiab] OR "Malaysia"[tiab] OR "Maldives"[tiab] OR "Mali"[tiab] OR "Marshall"[tiab] OR "Mauritania"[tiab] OR "Mauritius"[tiab] OR "Mexico"[tiab] OR "Micronesia"[tiab] OR "Moldova"[tiab] OR "Mongolia"[tiab] OR "Montenegro"[tiab] OR "Morocco"[tiab] OR "Mozambique"[tiab] OR "Myanmar"[tiab] OR "Burma"[tiab] OR "Namibia"[tiab] OR "Nepal"[tiab] OR "Nicaragua"[tiab] OR "Niger"[tiab] OR "Nigeria"[tiab] OR "Pakistan"[tiab] OR "Palau"[tiab] OR "Panama"[tiab] OR "Papua New Guinea"[tiab] OR "Paraguay"[tiab] OR "Peru"[tiab] OR "Philippines"[tiab] OR "Romania"[tiab] OR "Russian Federation"[tiab] OR "Russia"[tiab] OR "Rwanda"[tiab] OR "Samoa"[tiab] OR "Sao Tome and Principe"[tiab] OR "Principe"[tiab] OR "Senegal"[tiab] OR "Serbia"[tiab] OR "Sierra Leone"[tiab] OR "Solomon Islands"[tiab] OR "Somalia"[tiab] OR "South Africa"[tiab] OR "South Sudan"[tiab] OR "Sri Lanka"[tiab] OR "St. Lucia"[tiab] OR "St. Vincent and the Grenadines"[tiab] OR "Vincent"[tiab] OR "Sudan"[tiab] OR "Suriname"[tiab] OR "Swaziland"[tiab] OR "Syrian Arab Republic"[tiab] OR "Syria"[tiab] OR "Tajikistan"[tiab] OR "Tanzania"[tiab] OR "Thailand"[tiab] OR "Timor-Leste"[tiab] OR "Timor Leste"[tiab] OR "Togo"[tiab] OR "Tonga"[tiab] OR "Tunisia"[tiab] OR "Turkey"[tiab] OR "Turkmenistan"[tiab] OR "Tuvalu"[tiab] OR "Uganda"[tiab] OR "Ukraine"[tiab] OR "Uzbekistan"[tiab] OR "Vanuatu"[tiab] OR "Venezuela"[tiab] OR "Vietnam"[tiab] OR "Viet Nam"[tiab] OR "West Bank"[tiab] OR "Gaza"[tiab] OR "Yemen"[tiab] OR "Zambia"[tiab] OR "Zimbabwe"[tiab] OR "Rhodesia"[tiab] OR "Africa"[tiab] OR "Africa"[Mesh] OR "Central America"[Mesh] OR "Argentina"[Mesh] OR "Bolivia"[Mesh] OR "Brazil"[Mesh] OR "Colombia"[Mesh] OR "Ecuador"[Mesh] OR "Guyana"[Mesh] OR "Paraguay"[Mesh] OR "Peru"[Mesh] OR "Suriname"[Mesh] OR "Venezuela"[Mesh] OR "Mexico"[Mesh] OR "Asia, Central"[Mesh] OR "Russia"[Mesh] OR "Cambodia"[Mesh] OR "Timor-Leste"[Mesh] OR "Indonesia"[Mesh] OR "Laos"[Mesh] OR "Malaysia"[Mesh] OR "Mekong Valley"[Mesh] OR "Myanmar"[Mesh] OR "Philippines"[Mesh] OR "Thailand"[Mesh] OR "Vietnam"[Mesh] OR "Bangladesh"[Mesh] OR "India"[Mesh] OR "Afghanistan"[Mesh] OR "Iran"[Mesh] OR "Iraq"[Mesh] OR "Jordan"[Mesh] OR "Lebanon"[Mesh] OR "Syria"[Mesh] OR "Turkey"[Mesh] OR "Yemen"[Mesh] OR "Nepal"[Mesh] OR "Pakistan"[Mesh] OR "Sri Lanka"[Mesh] OR "China"[Mesh] OR "Mongolia"[Mesh] OR "Albania"[Mesh] OR "Bosnia and Herzegovina"[Mesh] OR "Bulgaria"[Mesh] OR "Kosovo"[Mesh] OR "Macedonia (Republic)"[Mesh] OR "Moldova"[Mesh] OR "Montenegro"[Mesh] OR "Republic of Belarus"[Mesh] OR "Romania"[Mesh] OR "Russia"[Mesh] OR "Serbia"[Mesh] OR "Developing countries"[Mesh]) |
| Avenir unit cost database | We reviewed the complete Avenir Unit Cost Repository to identify additional cost studies. We reviewed the titles and abstracts of all published studies in the database. |

## **Simulation model**

### Imputation of delivery volume

In cases when we had access to a complete sampling frame with information on the delivery volume at every site in the study population, we used the true distribution of delivery volumes in our simulation. In cases when the full sampling frame of delivery volumes was not available, we simulated a distribution of delivery volumes based on the distribution in the empirical sample. We fit a smooth kernel density to the distribution of delivery volumes in the original sample and then sampled randomly from this distribution, using a gamma kernel in order to ensure that the imputed delivery volumes were all positive. In addition to this approach, we also tried a simple bootstrap. We selected the kernel density approach as it allowed us to include values outside the range of the actual data. Figure S1 shows the kernel density fit to the empirical EPIC Ghana sample. In cases when the total number of delivery sites in the population was known, we generated populations with the true number of sites. Otherwise, we generated populations of 1000 delivery sites.

Figure S1: Kernel density of delivery volumes in EPIC Ghana sample

*
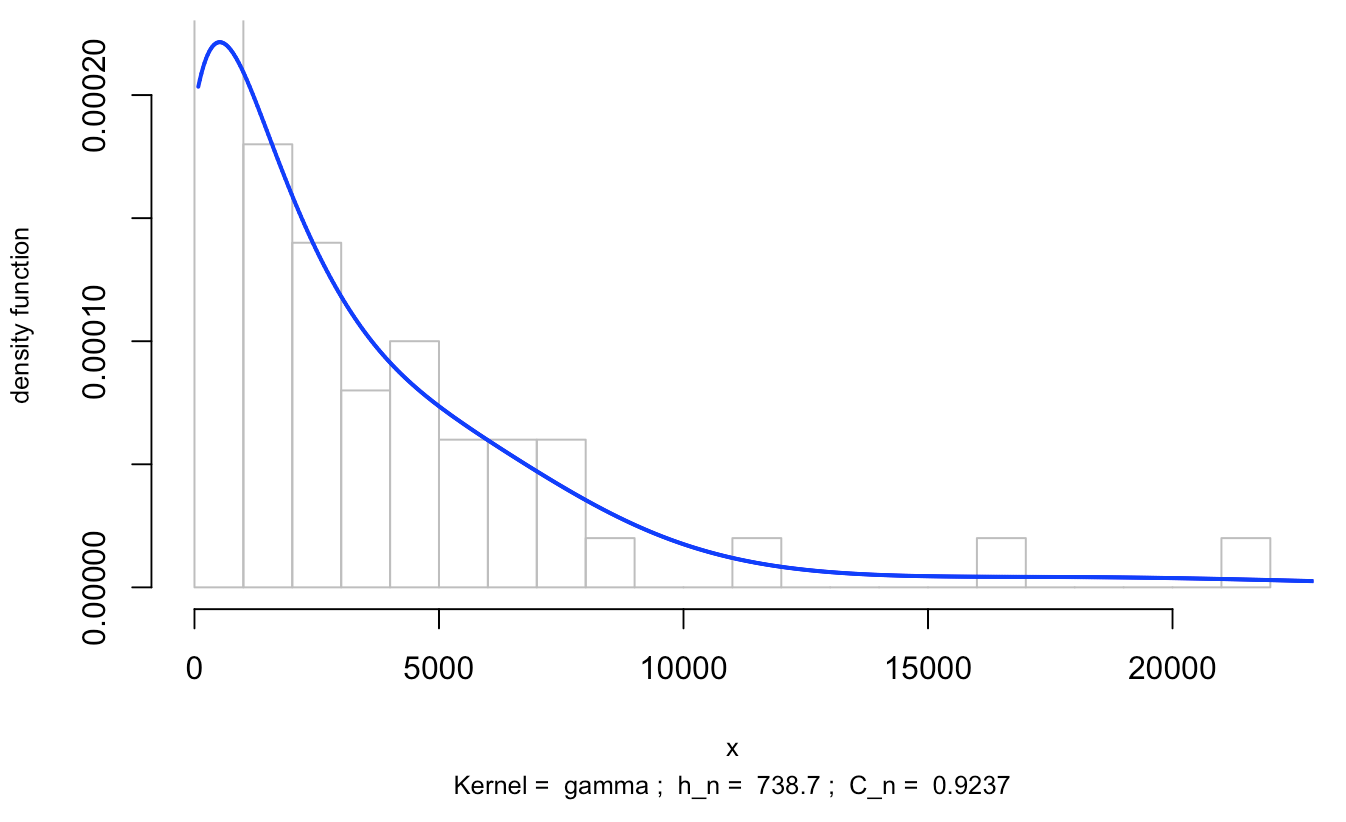
*

### Imputation of costs

To generate cost data, we used a semi-parametric approach, modelling the relationship between delivery volume and cost in the empirical dataset with thin plate regression splines. A conventional approach to incorporate variation into these estimates would be to draw from the sampling distributions assumed for the residuals (in this case, a gamma distribution). To relax this assumption, we instead sampled errors from the smoothed distribution of residuals from the cost-volume model. We did this by converting the residuals from the regression model into quantiles of a gamma function, fitting a kernel density to these quantiles using a beta kernel, sampling from this kernel density, and then converting the sampled quantiles back into residual values using the inverse gamma CDF. This approach allows us to include some outliers in the imputed cost dataset in a manner that is proportional to the presence of outliers in the sample dataset. As with the imputation of volumes, we also tried other methods including 2D kernel estimation and a simple bootstrap. We selected this approach because it gave us the best reproduction of the original relationships, while also allowing for values outside of those included in the original sample.

### Comparison of imputed populations with sample data

After imputing populations of site-level delivery volumes (for those datasets for which complete population information on delivery volumes was not available) and costs (for all datasets), we compared the imputed population distribution to the sample distribution to confirm that the imputed population realistically capitulated features of the sample distribution. Figure S2 compares the EPIC Ghana sample with the imputed population. The population volumes are from the sampling frame for the EPIC Ghana study; the population costs are imputed.

Figure S2: Comparison of EPIC Ghana sample and imputed population


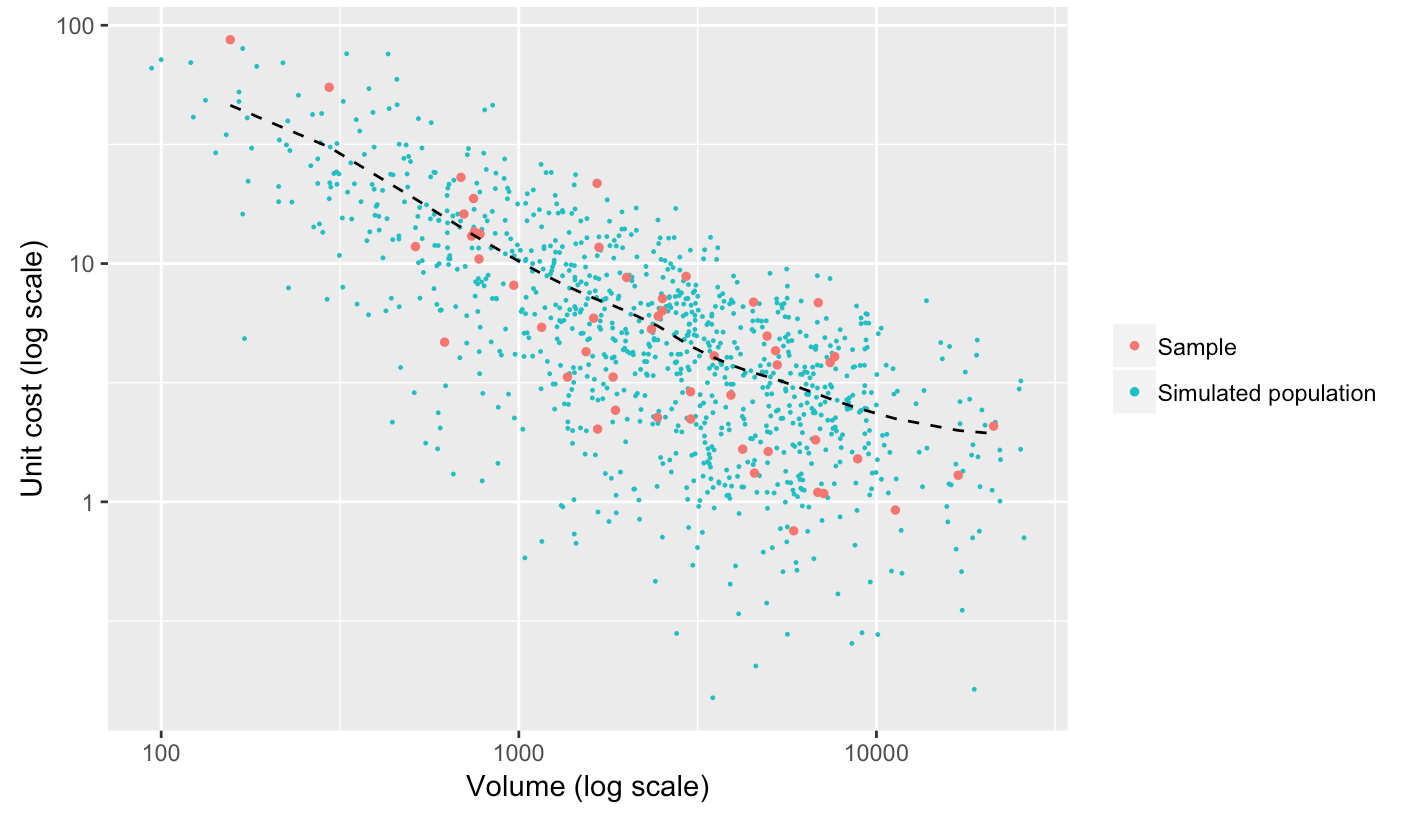


Notes: The x-axis is the volume of vaccine doses delivered at each site, on a log scale. The y-axis is the unit cost of delivering a vaccine dose, in log USD. The red dots are the health facilities in the empirical EPIC Ghana sample and the blue dots are the imputed population of health facilities. The dotted line shows the modelled relationship (using the thin plate regression spline model) between volume and unit costs.

# **Supplementary results**

## **Systematic review**

### Studies included

We screened 6774 titles and abstracts and identified 101 published studies that met the inclusion criteria for the systematic review (Figure S3). Bibliographic information for each of the included studies and extracted data are attached separately.

Figure S3: Flowchart of studies assessed for inclusion

We examined estimator use disaggregated by health domain (Table S1). We found that the simple average was reported in 32.6% of HIV costing studies, 30.8% of vaccination costing studies, and 57.1% of malaria costing studies.

Table S2: Estimator use by health domain

|  | **% of publications, by health domain** | | |
| --- | --- | --- | --- |
|  | **HIV** | **Vaccination** | **Malaria** |
| N | 43 | 13 | 7 |
| **Number of delivery sites (total, across all countries in study)** | **HIV** | **Vaccination** | **Malaria** |
| 5-9 | 25.6% | 7.7% | 14.3% |
| 10-14 | 14.0% | 15.4% | 14.3% |
| 15-19 | 18.6% | 15.4% | 42.9% |
| 20+ | 41.9% | 61.5% | 28.6% |
| **Summary estimator of central tendency** | **HIV** | **Vaccination** | **Malaria** |
| Simple average across sites | 32.6% | 30.8% | 57.1% |
| Average across sites, weighted by volume alone | 58.1% | 38.5% | 42.9% |
| Average across sites, weighted by other characteristics | 2.3% | 15.4% | 0.0% |
| Average across sites, weighted by volume and other characteristics | 0.0% | 30.8% | 0.0% |
| Median across sites | 25.6% | 7.7% | 28.6% |
| Simple average across individuals, sampled from multiple sites | 7.0% | 7.7% | 0.0% |
| Other | 18.6% | 7.7% | 14.3% |
| Not described | 14.0% | 0.0% | 0.0% |

*Publication may be counted twice if they fit into more than one of the categories listed under a given heading. For example, a publication may include both the simple average across sites and the average across sites, weighted by volume alone.

## **Simulation model**

### Estimator performance

Table S3 summarizes simulation findings across all datasets included in the simulation. These results were used to produce Figure 1. Measures of estimator performance are shown as a percentage of the true unit cost.

Table S3: Simulation findings across all included datasets

| **Estimator** | **Sample size** | **Measure of estimator performance** | **Maximum** | **Minimum** | **Mean** |
| --- | --- | --- | --- | --- | --- |
| Simple mean | 5 | absolute bias | 113.1 | 12.2 | 51.3 |
|  |  | standard deviation | 123.3 | 14.3 | 53.7 |
|  |  | RMSE | 167.3 | 18.8 | 75.2 |
|  | 10 | absolute bias | 111.4 | 12.2 | 51.0 |
|  |  | standard deviation | 86.5 | 10.1 | 37.8 |
|  |  | RMSE | 141.0 | 15.8 | 64.2 |
|  | 20 | absolute bias | 109.9 | 12.1 | 50.6 |
|  |  | standard deviation | 60.0 | 7.0 | 26.2 |
|  |  | RMSE | 125.2 | 14.0 | 57.5 |
|  | 40 | absolute bias | 110.4 | 12.2 | 50.6 |
|  |  | standard deviation | 41.7 | 4.9 | 18.3 |
|  |  | RMSE | 118.1 | 13.1 | 54.1 |
|  | 80 | absolute bias | 110.0 | 12.1 | 50.6 |
|  |  | standard deviation | 28.7 | 3.4 | 12.6 |
|  |  | RMSE | 113.7 | 12.5 | 52.3 |
| Median | 5 | absolute bias | 86.5 | 2.3 | 26.9 |
|  |  | standard deviation | 94.7 | 16.7 | 52.3 |
|  |  | RMSE | 118.7 | 19.0 | 60.6 |
|  | 10 | absolute bias | 82.2 | 2.0 | 24.0 |
|  |  | standard deviation | 61.2 | 11.6 | 34.5 |
|  |  | RMSE | 100.2 | 14.5 | 43.8 |
|  | 20 | absolute bias | 78.3 | 1.0 | 22.0 |
|  |  | standard deviation | 41.9 | 8.4 | 24.0 |
|  |  | RMSE | 88.8 | 11.7 | 34.2 |
|  | 40 | absolute bias | 76.2 | 0.8 | 21.2 |
|  |  | standard deviation | 30.4 | 6.0 | 16.8 |
|  |  | RMSE | 82.1 | 9.7 | 28.4 |
|  | 80 | absolute bias | 74.6 | 0.4 | 20.8 |
|  |  | standard deviation | 21.2 | 4.2 | 11.6 |
|  |  | RMSE | 77.6 | 5.9 | 24.9 |
| Weighted mean | 5 | absolute bias | 37.6 | 1.5 | 13.0 |
|  |  | standard deviation | 71.7 | 14.0 | 43.6 |
|  |  | RMSE | 80.9 | 14.0 | 45.9 |
|  | 10 | absolute bias | 20.1 | 0.6 | 7.0 |
|  |  | standard deviation | 50.5 | 10.1 | 30.6 |
|  |  | RMSE | 54.3 | 10.1 | 31.6 |
|  | 20 | absolute bias | 9.9 | 0.1 | 3.5 |
|  |  | standard deviation | 33.6 | 7.1 | 21.4 |
|  |  | RMSE | 35.0 | 7.1 | 21.8 |
|  | 40 | absolute bias | 5.2 | 0.1 | 1.8 |
|  |  | standard deviation | 22.6 | 5.0 | 15.0 |
|  |  | RMSE | 23.1 | 5.0 | 15.2 |
|  | 80 | absolute bias | 2.5 | 0.1 | 0.8 |
|  |  | standard deviation | 15.4 | 3.5 | 10.3 |
|  |  | RMSE | 15.6 | 3.5 | 10.3 |
| Calibration | 5 | absolute bias | 26.1 | 0.6 | 7.2 |
|  |  | standard deviation | 84.4 | 15.3 | 45.5 |
|  |  | RMSE | 84.5 | 15.3 | 46.3 |
|  | 10 | absolute bias | 19.7 | 0.0 | 4.1 |
|  |  | standard deviation | 47.9 | 10.7 | 26.8 |
|  |  | RMSE | 47.9 | 10.7 | 27.3 |
|  | 20 | absolute bias | 11.5 | 0.1 | 2.1 |
|  |  | standard deviation | 30.4 | 7.3 | 17.8 |
|  |  | RMSE | 30.4 | 7.3 | 18.1 |
|  | 40 | absolute bias | 5.1 | 0.0 | 1.1 |
|  |  | standard deviation | 21.1 | 5.1 | 12.4 |
|  |  | RMSE | 21.1 | 5.1 | 12.5 |
|  | 80 | absolute bias | 2.8 | 0.1 | 0.5 |
|  |  | standard deviation | 14.4 | 3.5 | 8.3 |
|  |  | RMSE | 14.4 | 3.5 | 8.4 |
| Regression | 5 | absolute bias | 5.71E+16 | 3.35E-01 | 2.86E+15 |
|  |  | standard deviation | 5.71E+18 | 1.79E+01 | 2.86E+17 |
|  |  | RMSE | 5.71E+18 | 1.80E+01 | 2.86E+17 |
|  | 10 | absolute bias | 54.1 | 0.1 | 7.7 |
|  |  | standard deviation | 808.7 | 9.9 | 110.2 |
|  |  | RMSE | 810.5 | 10.1 | 110.6 |
|  | 20 | absolute bias | 6.9 | 0.0 | 2.7 |
|  |  | standard deviation | 60.2 | 6.6 | 18.8 |
|  |  | RMSE | 60.6 | 6.9 | 19.1 |
|  | 40 | absolute bias | 5.2 | 0.2 | 2.0 |
|  |  | standard deviation | 23.5 | 4.6 | 11.7 |
|  |  | RMSE | 23.6 | 5.0 | 12.0 |
|  | 80 | absolute bias | 4.6 | 0.2 | 1.7 |
|  |  | standard deviation | 15.2 | 3.1 | 7.8 |
|  |  | RMSE | 15.2 | 3.6 | 8.1 |

Figure S4 shows how the bias in the simple mean estimator varies as a function of the coefficient of variation in delivery volume and the elasticity of unit costs with respect to delivery volume.

Figure S4: Bias in the simple mean estimator as a function of variation in delivery volume and elasticity of unit costs with respect to delivery volume


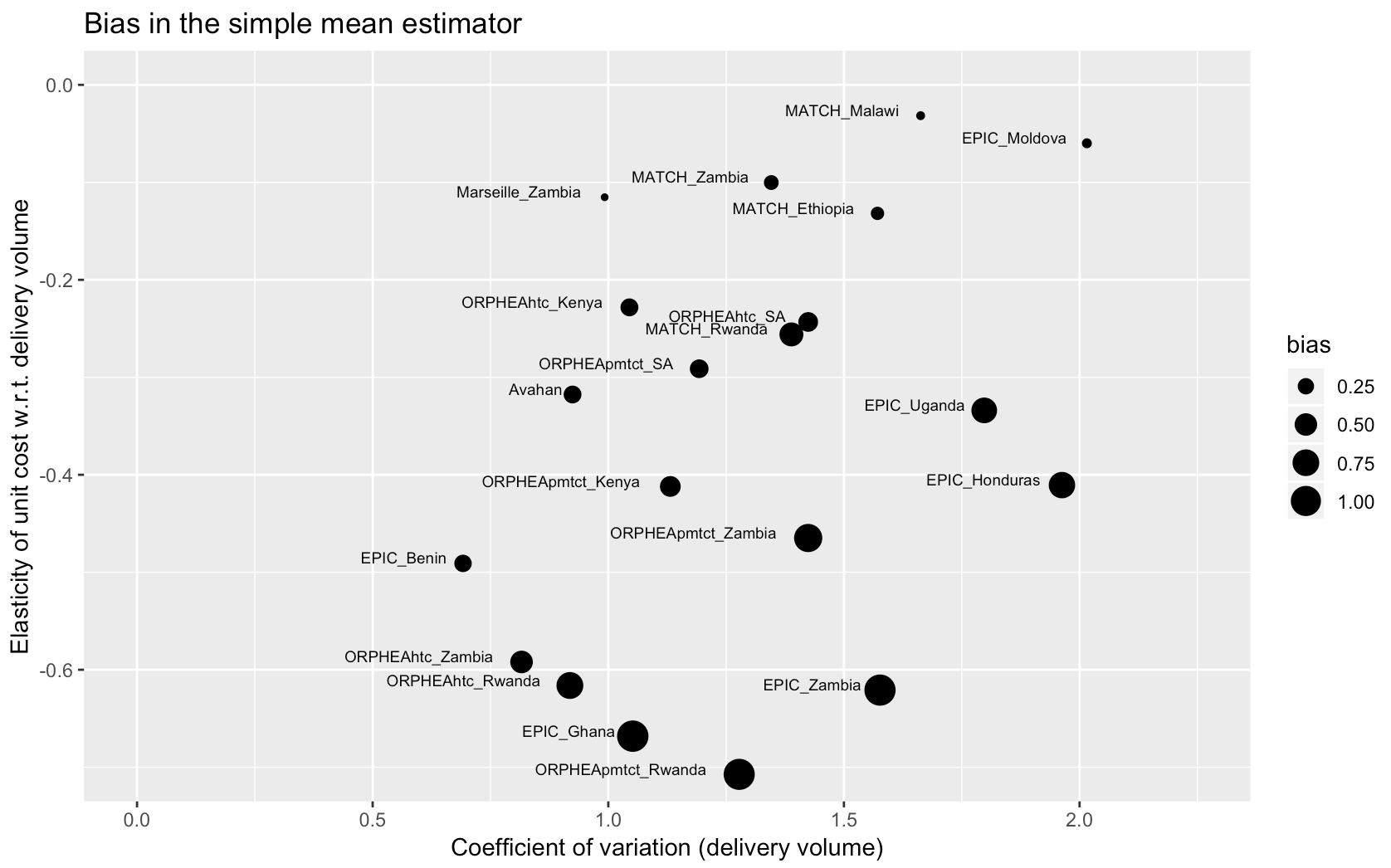


Notes: Each point on the figure represents one simulated population of healthcare delivery sites. The label corresponds to the empirical dataset used to simulate that population. The y-axis is the elasticity of unit cost with respect to delivery volume in the population. The x-axis is the coefficient of variation of delivery volume in the population. The size of the point corresponds with the amount of bias in the simple mean estimator in this population, with studies of sample size 80, presented as a proportion of the true unit cost.

*Findings from EPIC Honduras imputed dataset*

In addition to the pooled analysis described in the main paper, we analyzed results from each of the simulated populations independently. Results from the simulation in the EPIC Honduras sample are described below and summarized in Figures S5 and S6. These findings are consistent with our overall findings from the pooled analysis.

Focusing first on the RMSE, we see that the calibration estimator performed the best in small samples (with an RMSE of 50.1% of the true unit cost), followed by the weighted mean (56.3%), and then the median (74.2%), the simple mean (90.5%), and finally the regression estimator (82697.5%). The regression estimator had the lowest RMSE of all of the estimators included in the simulation study for samples of size 10 and above.

All of the estimators included in the simulation are biased in samples of five sites. For some estimators (the calibration estimator, regression estimator, and weighted mean), this bias decreases as the sample size increases; for others (the simple mean and median), it does not. The calibration estimator has an upward bias of 14.2% of the true unit cost in samples of five sites. This bias decreases to 1.6% of the true unit cost in samples of 80 sites. The volume-weighted has an upward bias of 26.9% of the true unit cost in samples of 80 sites and 2.5% of the true unit cost in samples of 80 sites. The median has an upward bias of 52.3% of the true cost in samples of five sites; this decreases slightly as the sample size increases but is still 45.1% of the true cost in samples of 80 sites. The simple mean has a large upward bias of 73.3% in samples of five sites that remains nearly constant as the sample size increases: in samples of 80 sites, this bias is still very high at 73.0% of the true unit cost.

Figure S5: Boxplot comparison of estimator performance in imputed population based on EPIC Honduras cost dataset


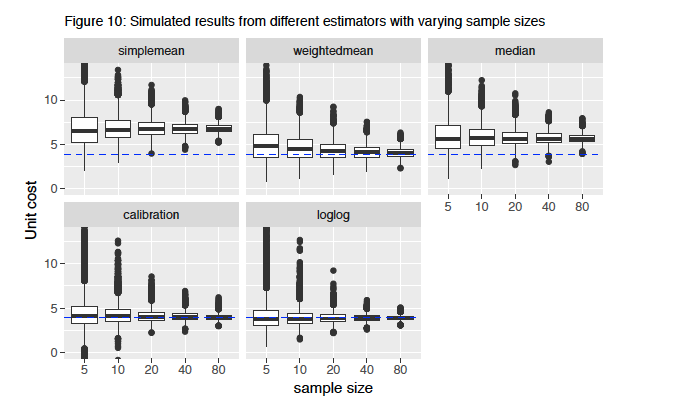


Notes: The x-axis is sample size used in the simulation, and the y-axis is the unit cost. The blue line shows the true unit cost in the population. The box plots show the inter-quartile range and median cost estimate across simulations of a given sample size using each estimator. The panels show findings for each of the five estimators evaluated in the study (the simple mean, weighted mean, median, calibration estimator, and regression estimator with a log-log specification).

Figure S6: Comparison of estimator performance in imputed population based on EPIC Honduras cost dataset (log scale)


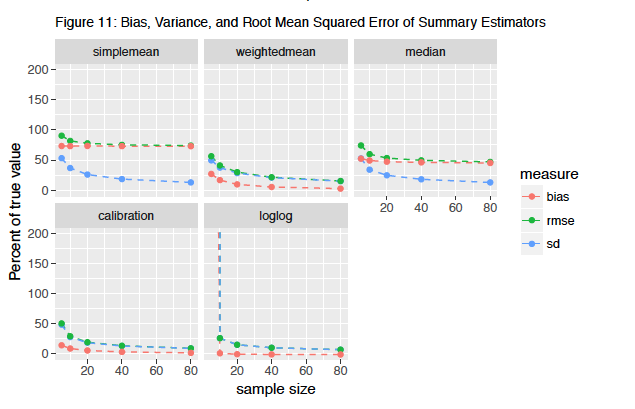


Notes: The x-axis is sample size used in the simulation, and the percent of the true cost, shown on the log scale. Thus, a bias of 100% indicates that the estimate was twice as large as the true value. The red line shows bias. The green line shows root mean squared error (RMSE). The blue line shows the standard deviation. The panels show findings for each of the five estimators evaluated in the study (the simple mean, weighted mean, median, calibration estimator, and regression estimator with a log-log specification).

2.2.2. Sensitivity analyses: different sampling approaches

We conducted sensitivity analyses to examine how our results changed with stratified sampling or sampling proportional to site size. Both sensitivity analyses were conducted using the imputed EPIC Honduras population dataset.

To implement stratified sampling, we grouped delivery sites according to type (as done in the original EPIC study). Since the full sampling frame was available for the EPIC Honduras study, we know the site type for all sites in the imputed population. There are three types of sites in this dataset (Hospitals, Cesamos, and Cesars). Facility type is associated with size. These types are based on facility size. To simplify the analysis, we drew repeated samples of size 6, 12, 24, 48, 96 (rather than 5, 10, 20, 40, and 80) so that the number would be divisible by three. We included equal numbers of each facility type in each sample (e.g. samples of 6 included 2 Hospitals, 2 Cesamos, and 2 Cesars). The simple mean was estimated by calculating the simple mean of unit costs within each stratum in the sample, and then weighting these means according to the distribution of the three strata in the population. The weighted mean was estimating by calculating the volume-weighted mean of unit costs within each stratum in the sample, and then weighting these means according to the distribution of the three strata in the population. The calibration estimator was implemented by taking strata into account in the inverse probability weights. Finally, the regression estimator was implemented by including the facility type as a categorical predictor in the regression model.

As shown in Figure S7, our conclusions do not change significantly with stratified sampling. The sample mean still maintains a large upwards bias as the sample size increases: with samples of 96 sites, the simple mean exhibits an upward bias of 73.5% of the true unit cost. The median, weighted mean, regression estimator, and calibration estimator all have increased precision with stratified sampling. For example, the standard deviation of the weighted mean with simple random sampling and 80 sites is 15.0% of the true unit cost, while the standard deviation of the weighted mean with stratified sampling and 48 sites is 14.7% of the true unit cost.

Figure S7: Comparison of estimator performance in imputed population based on EPIC Honduras cost dataset (log scale) with stratified sampling


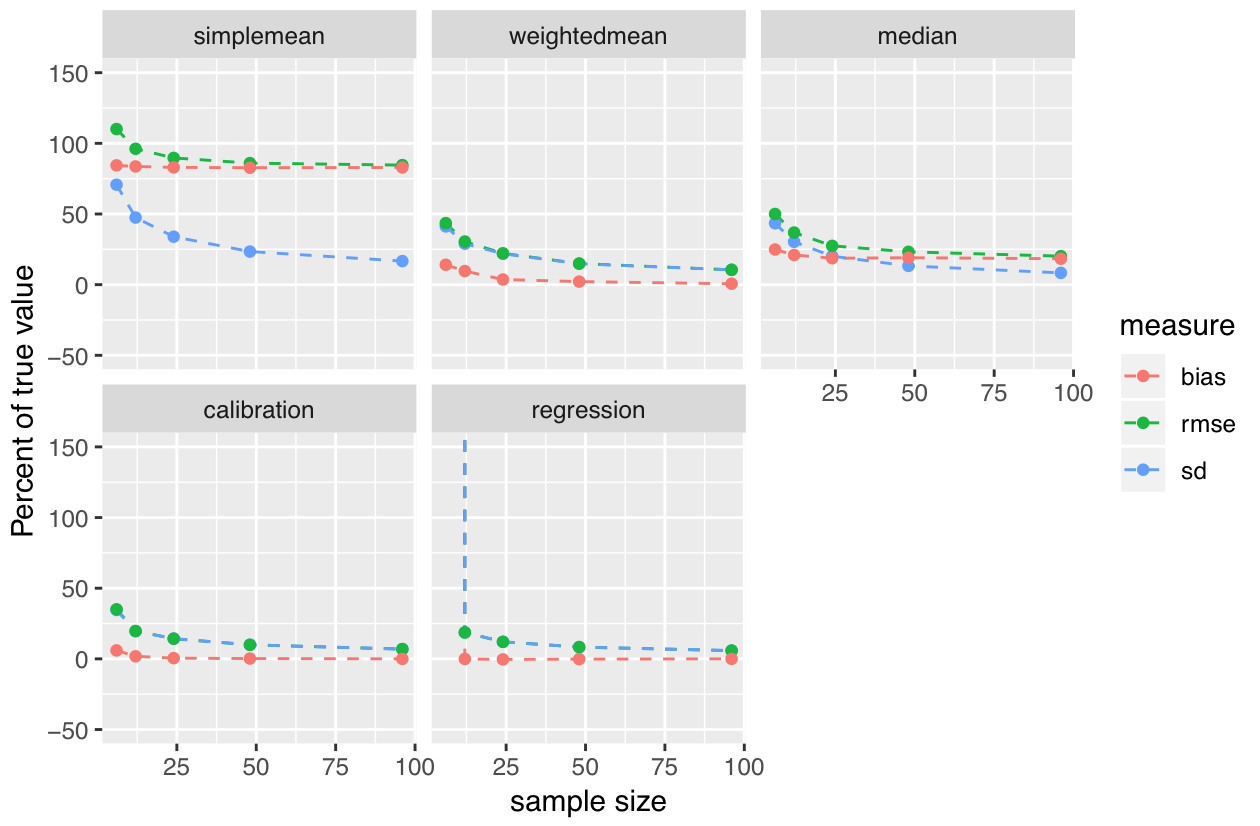


Notes: The x-axis is sample size used in the simulation, and the percent of the true cost, shown on the log scale. Thus, a bias of 100% indicates that the estimate was twice as large as the true value. The red line shows bias. The green line shows root mean squared error (RMSE). The blue line shows the standard deviation. The panels show findings for each of the five estimators evaluated in the study (the simple mean, weighted mean, median, calibration estimator, and regression estimator). Sampling is stratified by health facility type.

1. To implement sampling proportional to size, we conducted weighted sampling where the probability of a site being selected into the sample was proportional to the delivery volume at that site. The five estimators were all calculated using the same approach as in the main analysis. Sampling was done with replacement so that the probability with which a given site appeared in a sample (across multiple samples) could truly be proportional to its size.
2. As shown in Figure S8, under sampling proportional to size, the simple mean no longer has an upward bias. Essentially, with this sampling approach, the volume-based weighting occurs at the sampling stage rather than the analysis stage.

Figure S8: Comparison of estimator performance in imputed population based on EPIC Honduras cost dataset with sampling proportional to size


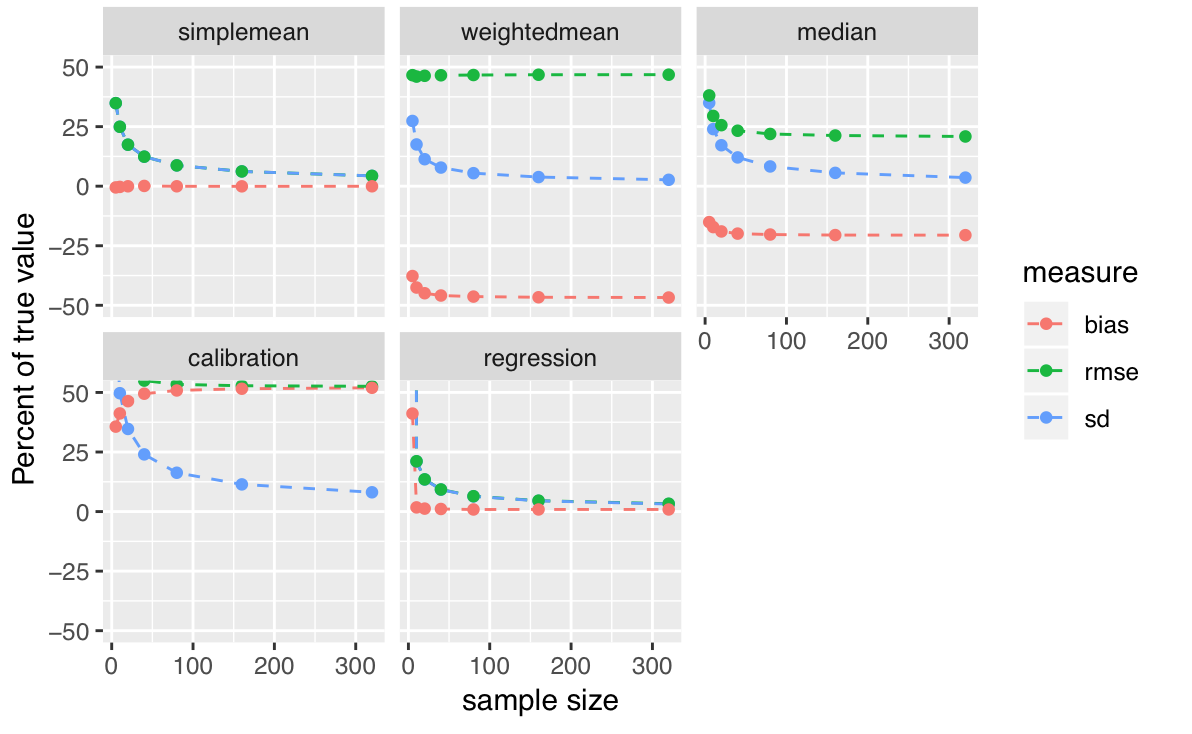


Notes: The x-axis is sample size used in the simulation, and the percent of the true cost. Thus, a bias of 100% indicates that the estimate was twice as large as the true value. The red line shows bias. The green line shows root mean squared error (RMSE). The blue line shows the standard deviation. The panels show findings for each of the five estimators evaluated in the study (the simple mean, weighted mean, median, calibration estimator, and regression estimator). Sampling is proportional to size (i.e. delivery volume).
